# Acute left ventricular dysfunction secondary to right ventricular septal pacing in a woman with initial preserved contractility: a case report

**DOI:** 10.1186/1752-1947-5-524

**Published:** 2011-10-25

**Authors:** Sana Ouali, Soufiene Azzez, Slim Kacem, Afef Lagren, Elyes Neffeti, Rim Gribaa, Fahmi Remedi, Essia Boughzela

**Affiliations:** 1 Department of Cardiology. Sahloul Hospital, Sousse, Tunisia

## Abstract

**Introduction:**

Right ventricular apical pacing-related heart failure is reported in some patients after long-term pacing. The exact mechanism is not yet clear but may be related to left ventricular dyssynchrony induced by right ventricular apical pacing. Right ventricular septal pacing is thought to deteriorate left ventricular function less frequently because of a more normal left ventricular activation pattern.

**Case presentation:**

We report the case of a 55-year-old Tunisian woman with preserved ventricular function, implanted with a dual-chamber pacemaker for complete atrioventricular block. Right ventricular septal pacing induced a major ventricular dyssynchrony, severe left ventricular ejection fraction deterioration and symptoms of congestive heart failure. Upgrading to a biventricular device was associated with a decrease in the symptoms and the ventricular dyssynchrony, and an increase of left ventricular ejection fraction.

**Conclusion:**

Right ventricular septal pacing can induce reversible left ventricular dysfunction and heart failure secondary to left ventricular dyssynchrony. This complication remains an unpredictable complication of right ventricular septal pacing.

## Introduction

Right ventricular apical (RVA) pacing results in abnormal left ventricular (LV) electrical and mechanical activation and is associated with an increased risk of developing heart failure [1-3]. Right ventricular septal (RVS) pacing has been introduced to avoid this apparent and unpredictable complication of RVA pacing, because this pacing site appears to deliver a more physiological electrical activation of both ventricles, visible with a shorter paced QRS complex, than with RVA pacing [4,5].

We report the case of a 55-year-old Tunisian woman with preserved ventricular function, implanted with a dual-chamber pacemaker for complete atrioventricular block. RVS pacing induced a major ventricular dyssynchrony, severe left ventricular ejection fraction deterioration and symptoms of congestive heart failure. Upgrading to a biventricular device was associated with a decrease in the symptoms and ventricular dyssynchrony, and increased left ventricular ejection fraction (LVEF).

### Case presentation

**Figure 1:**
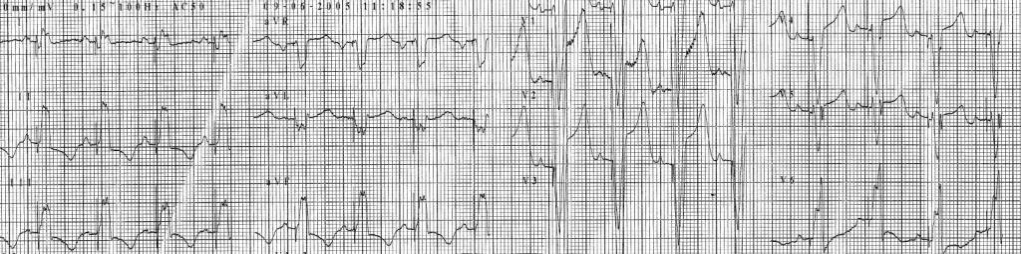
**Twelve lead ECG after DDD pacemaker implantation**. Note the QRS morphology with negative deflection of lead I and positive initial R-waves of the paced ventricular complex in lead aVF.

**Figure 2:**
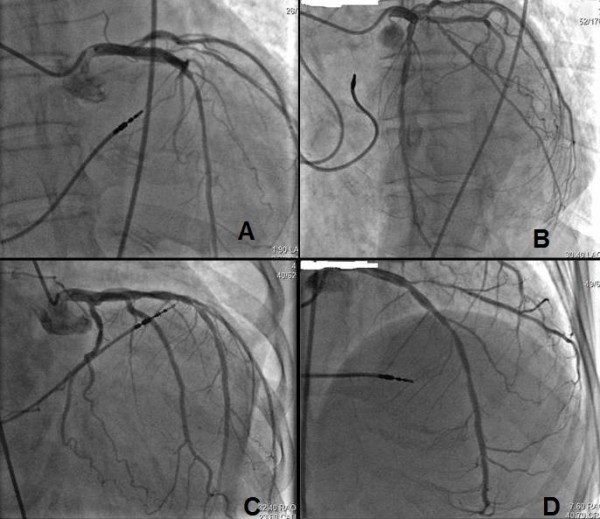
**Coronary angiogram of the left coronary artery**. Fluoroscopic images at an anteroposterior (AP, Panel A), left anterior oblique (LAO, Panel B), right anterior oblique (RAO, Panel C) and cranial (Cranial, Panel D) projection, showing the position of the active ventricular pacing lead at the RV septal region (arrow). Note the proximity of the septal lead tip to the left anterior descending artery.

A 55-year-old Tunisian woman presented with syncope. An electrocardiogram (ECG) upon admission showed complete heart block with a narrow QRS complex (<120 ms) and an escape ventricular rate of 45 bpm. Our patient's medical history included arterial hypertension. She did not have diabetes mellitus, and had no family history of coronary artery disease. A two-dimensional echocardiography showed normal LV function with a 60% EF, the absence of significant valvulopathy and no regional wall motion abnormalities or pulmonary artery hypertension. A conventional dual chamber pacemaker (Medtronic; Sensia SEDR01, US) was implanted with the right ventricular (RV) lead positioned to her RV septum. The septal position was confirmed by fluoroscopic images; defined as a leftward orientation of the lead confirmed by 40° left anterior oblique projection [6]. The electrocardiographic criteria were defined as a negative deflection of lead I and positive initial R-waves of the paced ventricular complex in lead ventricular fibrillation (VF) [7]. The pacemaker was programmed in a DDD mode with lower rate of 50 bpm and upper tracking rate of 120 bpm. An ECG before discharge showed atrial synchronized ventricular pacing with a rate of 80 bpm and QRS duration of 160 ms (Figure 1). Echocardiographic examination two days after pacemaker implantation demonstrated a normal LV function (55%), a LV end-diastolic volume (LVEDV) of 84 mL, the absence of significant valvulopathy and an aortic pre-ejection period (PEP) of 160 ms. A ventricular dyssynchrony (80 ms between septal and lateral electromechanical delays) was also measured with tissue Doppler imaging (TDI). The ratio of E (peak transmitral flow velocity in early diastole) to Ea (peak early diastolic myocardial velocity) velocity (E/Ea) was estimated at 5.25. Our patient was readmitted seven months later with six days of progressive dyspnea (New York Heart Association (NYHA) class IV). Echocardiography showed severe LV akinesis, a depressed LVEF (28%), a LVEDV of 153 mL, the presence of significant mitral and tricuspid regurgitations (grade II-III), an aortic PEP of 170 ms, pulmonary artery hypertension (50 mmHg) and an E/Ea ratio of 6. Her troponin level was not raised. Coronary angiography revealed the absence of significant obstructive epicardial coronary artery disease (Figure 2) and left ventriculography demonstrated depressed LVEF (25%).

**Figure 3:**
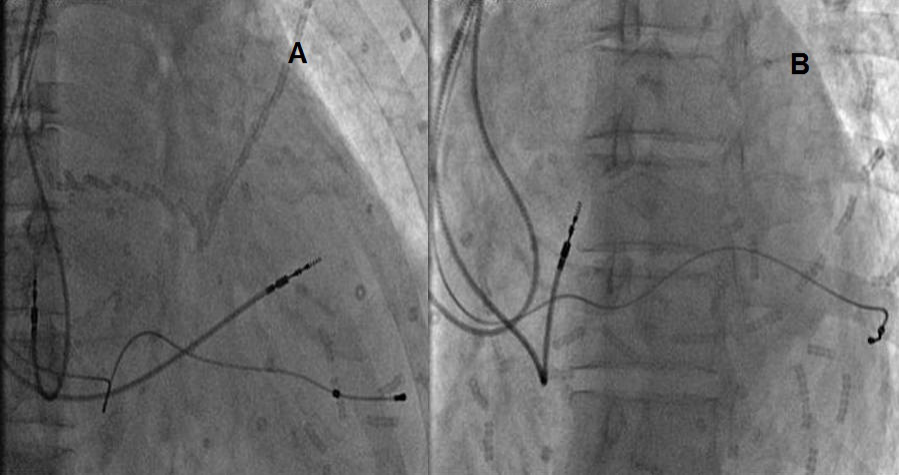
**Anteroposterior (AP, Panel A) and left anterior oblique (LAO, Panel B) fluoroscopic projections showing leads position after CRT**.

**Figure 4:**
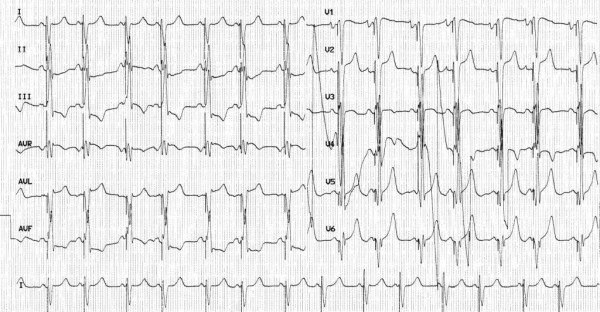
**Twelve lead ECG after CRT**.

Despite instauration of optimal medical therapy, our patient remained at NYHA functional class III. She was upgraded to a cardiac resynchronization therapy (CRT)-device with implantation of a lateral left ventricular lead (Figure 3). After one month of CRT, symptoms and exercise tolerance improved markedly from NYHA class III to class II. A twelve-lead ECG showed QRS shortening after CRT (Figure 4).

After one month, an echocardiography showed a decrease in the aortic PEP (130 ms), LV reverse remodeling, with a reduction of the LVEDV from 153 mL to 135 mL, and significant improvement in LVEF to 40%. She had no symptoms of heart failure or syncope afterwards and device interrogation showed that her cumulative biventricular pacing was 100%.

## Discussion

Pacing from RVS sites has been suggested as an alternative to RVA pacing in an attempt to avoid long-term adverse consequences on LV function [4]. This case illustrated the rare phenomenon of rapid development of heart failure and dramatic decrease of LVEF after short-term RVS pacing for a complete atrioventricular block in a woman with initially preserved LVEF. This case also showed the reversible nature of RVS pacing-induced heart failure, and that it may be related to the reversible LV dyssynchrony induced by RVS pacing, as demonstrated by TDI and an aortic PEP of 160 ms. There seems to be no other cause to account for the heart failure in this woman except for RVS pacing.

There is an increasing body of literature in which the authors investigate the acute and chronic effects of RVS pacing on electrical and mechanical synchrony, systolic and diastolic ventricular function and cardiovascular morbidity and mortality. Alternative RV pacing sites appear advantageous when compared to RVA pacing but their superiority has not been uniformly proven.

Ten Cate *et al. *[8] have demonstrated that acute abnormal LV activation either forms RVA or RV outflow tract (RVOT) pacing, resulting in an acute diminished LV function as assessed with echocardiographic wall motion score, traced LVEF, electromechanical delay and regional longitudinal LV strain. The authors have suggested that any RV pacing sites can negatively affect LV function and that readily available and non-invasive echocardiographic techniques are not helpful to guide the selection of the individual optimum pacing site during implantation. In the same way, Ng *et al. *[9] demonstrated that standard fluoroscopic and electrocardiographic implantation techniques for RVS pacing resulted in a heterogenous group of different pacing sites. They found that the patients with RVS pacing had a lower LVEF, lower circumferential strain and greater circumferential dyssynchrony than those patients with RVA pacing, despite achieving a narrower QRS complex. They concluded that these detrimental effects associated with RVS pacing might have resulted from the heterogeneity of the real pacing sites included under the umbrella of the RVS pacing concept.

In patients with standard indications for pacing, the prediction of heart failure is difficult and the exact mechanism of RV pacing-related heart failure is not clear but may be related to LV dyssynchrony induced by RV pacing [10]. The best treatment option for these patients remains to be determined. CRT seems to be superior to RV pacing in patients with either impaired [11] or preserved LV systolic function [12] and standard pacing indication.

The Pacing to Avoid Cardiac Enlargement study [12] showed that the mean LVEF declined by almost seven percentage points (from 61.5 ± 6.6% to 54.8 ± 9.1%) in the first year of RVA pacing in patients with a normal ejection fraction. Among nine patients in whom the LVEF decreased to less than 45% at 12 months, eight (89%) were in the RV pacing group. The authors suggest that the ejection fraction could decrease rapidly in vulnerable patients and that these patients might benefit even more from biventricular pacing [12].

## Conclusion

RVS pacing can induce reversible LV dysfunction and heart failure secondary to LV dyssynchrony. This remains an unpredictable complication of RV pacing. It should be highlighted that not all patients develop LV dyssynchrony and new onset heart failure after RV pacing. Therefore, early predictive factors [13-15], such as dyssynchrony at the time of implantation, paced QRS width, age, presence of atrial fibrillation, concomitant coronary artery disease, compromised LVEF or antibody status, should be further evaluated. These factors may reveal the patients who are more prone to LV function deterioration and who are consequently better candidates for biventricular pacing.

## Consent

Written informed consent was obtained from the patient for publication of this case report and any accompanying images. A copy of the written consent is available for review by the Editor-in-Chief of this journal.

## Competing interests

The authors declare that they have no competing interests.

## Authors' contributions

SO was the major contributor in writing the manuscript. All authors read and approved the final manuscript.
